# Psychiatric morbidity in geriatric people

**DOI:** 10.4103/0019-5545.31596

**Published:** 2006

**Authors:** Prashant Tiple, S.N. Sharma, A.S. Srivastava

**Affiliations:** * Senior Lecturer Department of Psychiatry, MGIMS, Sevagram, Wardha; ** Retired Professor and Head Department of Psychiatry, IMS, BHU, Varanasi (UP); *** Reader Department of Psychiatry, IMS, BHU, Varanasi (UP)

**Keywords:** Geriatric, depression, hypertension, social support

## Abstract

**Background:**

The number of geropsychiatric patients is increasing but sufficient work has not been done in this area in many parts of India.

**Aim:**

This study explored the sociodemographic profile and clinical characteristics of patients aged 60 years and above, attending the psychiatric services of Institute of Medical Sciences and geropsychiatric patients of Mumukshu Bhavan (old age home) in Varanasi from September 1998 to September 1999.

**Methods:**

For the screening of psychiatric patients at Mumukshu Bhavan the Indian Psychiatric Survey Schedule was used. DSM-IV criteria were used for the diagnosis of patients and Chi-square test with Yate correction and Z-test were used for statistical analysis.

**Results:**

Depressive disorders were the most common psychiatric illnesses. Many patients had associated physical illnesses and among them hypertension was the most common. Family jointness was adequate for most of the patients. Objective social support was moderate for the majority of patients but perceived social support was poor. Patients of Mumukshu Bhavan perceived their social support to be either moderate or good.

**Conclusion:**

Depressive disorder was the most common psychiatric illness and among the physical illnesses hypertension was the commonest. People living in the old age home felt better than those who lived with their children's family.

## INTRODUCTION

Of the total world population of 4.37 billion, about 250 million are over the age of 65 years.^1^ The population of India increased from 685 million (geriatric group 6.4%) in 1981 to 846 million (geriatric group 7.5%) in 1991. The life expectancy of an average Indian has increased from 54 years in 1981 to 64.6 years in 2002.^2^ According to Sharma the population of people aged 60 years or above is likely to increase to 18.4% of the total population by the year 2025.^3^ Improved healthcare promises longevity but social and economic conditions such as poverty, break-up of joint families and poor services for the elderly pose a psychiatric threat to them.^4^

The modern era of geriatric psychiatry began in the early part of the nineteenth century with the differentiation of senile dementia, arteriosclerotic dementia and presenile psychosis. The need for research in geropsychiatry has increased because of the growth in size of the elderly population. The large geriatric population has an equally high psychiatric morbidity. Various studies have been carried out in India to estimate the prevalence rates, notable among which are Dubey (23.3/1000 population) and Nandi *et al. 33%.*^5^,^6^ Ramchandran *et al.* found that psychiatric disorders were present in 35% of the elderly population, out of which the rates of depression and schizophrenia were found to be 240 and 10 per 1000 population, respectively.^7^

Nielsen studied mental illness in old age in a Danish population and found that a low prevalence of psychiatric disorders was associated with those living with a spouse; a high rate was seen in those living with relatives or children, and the highest was for those living in an old age home.^8^

Community surveys of psychiatric morbidity by Dubey^5^ and Verghese *et al.*^9^ in India revealed a peak incidence of psychiatric disorders in middle age and a fall in old age.

Using DSM-IV diagnostic criteria Martha *et al.* found that 13.5% of newly admitted elderly home care patients suffered from major depression.^10^ Ritchie *et al.* found a lifetime prevalence of 26.5% and 30% for major depression and anxiety disorders in geropsychiatric patients, respectively.^11^ Judith *et al.* found that instrumental social support provides marginal protection against worsening performance on instrumental activities of daily living, which were primarily a function of baseline depression severity.^12^ McNulty *et al.* found that in older patients with schizophrenia co-morbid cognitive impairment and physical illness needed management by adequately resourced specialized old age psychiatric services.^13^

Kautilya, the ancient law-giver of India, had stated that it was obligatory for the State to care for the older members of its community.^14^ The aged were assigned the last two stages of life among the four described since ancient times, the so called *Varnashramas:* (i) the student *(brahmacharya),* (ii) the householder *(grihastha),* (iii) the ascetic *(vanprastha),* and (iv) the forest dweller *(sanyas).* All have to pass through these stages. An attitude of reverence for the aged is looked upon as a virtue. The elders are given a fairly high rank in the family hierarchy and their blessings and decisions on important occasions are frequently sought.^15^

With an increase in the geriatric population and the expected decline in the proportion of middle-aged people, who are the caregivers for both the geriatric and paediatric population, the burden on this group is likely to mount leading to newer and unforeseen problems.

This study was undertaken with the following aims:

- To study the various types of psychiatric illnesses among
  —elderly patients visiting the psychiatry OPD of S.S. Hospital, BHU, Varanasi
  —referred cases from the geriatric clinic of S.S. Hospital, BHU, Varanasi
- To screen and study patients with psychiatric problems among the elderly residents of Mumukshu Bhavan (old age home)
- To understand the pattern of associated physical illness in geropsychiatric patients.

## METHODS

The present study was carried out in the Department of Psychiatry, Geriatric Clinic (a division of the Department of General Medicine) and Mumukshu Bhavan, from September 1998 to September 1999. The patients who came to the psychiatric OPD of the Department of Psychiatry and Geriatric Clinic of General Medicine on fixed days were studied.

A door-to-door screening of geriatric persons was done in Mumukshu Bhavan to detect geropsychiatric patients. Those suspected of having a psychiatric problem were interviewed in detail for the detection of psychiatric illnesses and associated physical illnesses.

The number of subjects studied in the Psychiatric OPD, cases suspected of having a psychiatric illness referred from the Geriatric Medicine OPD and residents of Mumukshu Bhavan were 92, 8 and 68, respectively.

*Inclusion criteria:* All subjects who were 60 years and above and were willing to participate in the study.

Written, informed consent was taken from the subjects. In the case of patients who were not able to give consent because of their psychiatric illness, it was taken from their relatives.

The participants in the study were divided into four groups.

- *Group A:* Geriatric subjects who visited the psychiatric OPD (*n*=92). Out of these 92 cases, 8 had only a physical illness and were excluded. The remaining 84 patients were studied.
- *Group B:* Geriatric subjects suspected to have psychiatric illness and referred from the Geriatric Clinic (*n*=8).
- *Group C:* Householders living in Varanasi in the hope of attaining ‘moksha’ and were paying for their boarding and lodging. They had occasional contact with their family members (*n*=45).
- *Group D:* Ascetics who had left their families earlier and their daily living costs were borne by Mumukshu Bhavan (*n*=23).

All subjects in groups A and B and suspected psychiatric cases identified after administration of the Indian Psychiatric Survey Schedule^16^ in groups C and D were interviewed according to a proforma, which included sociodemographic details, mode of referral, chief complaints, precipitating factors, history of present illness, history of past illness, family history, personal history, details about pre-morbid personality, physical and mental status examination. The DSM-IV criteria were used for diagnosis. The family jointness and social support were measured by the Family Jointness scale and Social Support scale, respectively.^17^,^18^

Family Jointness scale by Agrawal *et al.* was administered to assess the family jointness of our geriatric patients.^17^ The scale has three categories: (i) financial jointness, (ii) jointness in living, and (iii) jointness in decision-making. Each category has four items for each of which the options were ‘Yes’ and ‘No’. A ‘yes’ response on items 1, *2, 3* and *4* is scored as 1, *2, 3* and *4,* respectively.

In the original Family Jointness scale ‘jointness in living’ is scored as 1 if the spouse lives independently from members of the parental family.^17^ In this, a slight modification was made to suit the geriatric patients under study. In the modified Family Jointness scale the term ‘parental families’ was replaced by the term ‘offspring's families’ and the term ‘spouse’ by ‘case’ and ‘spouse (if alive)’. This modification was done because the original scale was made for the middle-aged population, where spouses were getting support from parental families. As spouses of some of the geriatric patients were likely to have expired due to old age the word ‘spouse’ in the original scale was replaced by ‘case’ and ‘spouse (if alive)’.

### Social support

The Social Support scale devised by Aneshensel *et al.* was used to measure the social support of patients. Two measures of social support were assessed: (i) an objective measure of the number of close relationships, and (ii) a subjective measure of perceived social support.

#### Objective social support

To ascertain the Objective Social Support score, respondents were asked for the number of close relatives and close friends they had, with ‘close’ described as ‘people you could feel at ease with, could talk about private matters and call for help’.

The scoring for objective social support was done as follows:

**Table:** 

*Score*	*Objective social support*
1 Absent	Having no close friend, no close relative or spouse
2 Poor	Having one or more than one close friend
3 Moderate	Having one close relative and/or one or more than one close friend
4 Good	Healthy spouse with close relatives and/or close friend (close relatives include offspring of the person under study).

#### Perceived social support

To ascertain the Perceived Social Support score, two types of support systems were assessed: (i) socioemotional support (e.g. thoughtfulness, understanding), and (ii) instrumental help (e.g. assistance with work or with problems).

The respondents were asked ‘How often during the past two months had someone provided them with help?’ Slight modification was done in scoring because there was no division in the original scoring between score 1 (not at all) and score 4 (very often), whereas in the practical set-up intermediate ranges of (i) socioemotional support and (ii) instrumental help were received.

Socioemotional support and instrumental help were scored separately as follows:

**Table:** 

*Scores*	*Items*
1	Never
2	Once in two months
3	More than once in two months
4	Whenever required

Perceived Social Support scores were arrived at by adding the scores of socioemotional support and instrumental help.

**Table:** 

*Score*	*Perceived social support*
Upto to 2	Absent
3–4	Poor
5–6	Moderate
7–8	Good

Chi-square test with Yate correction and Z-test were used for statistical analysis.

## RESULTS

Out of 92 subjects who attended the Psychiatry OPD, 84 were found to be psychiatrically ill. The remaining 8 patients were suffering from physical illnesses and had been brought to the psychiatric OPD due to lack of awareness of their attendants regarding the type of illness. All the 8 patients referred from the Geriatric Clinic were found to be psychiatrically ill.

At the Mumukshu Bhavan in group C, out of 45 house-holders, 17 were found to be psychiatrically ill and in group D out of 23 ascetics, 6 were found to be psychiatrically ill.

The majority of subjects in groups A, B and C fell in the age group of 60–69 years. Group C comprised mainly males, while in group D all were males (Table 1).

**Table 1:** Age and sex distribution in groups A, B, C and D

	Group A (n=84)		Group B (*n*=8)		Group C (*n*=17)		Group D (*n*=6)	
				
	*n*	%	*n*	%	*n*	%	*n*	%
Age (years)								
60-69	78	(92.85)	7	(87.5)	12	(70.58)	3	(50)
70-79	5	(5.95)	1	(12.5)	4	(23.52)	2	(33.33)
80 and above	1	(1.19)	0	(0)	1	(5.88)	1	(16.66)
Sex								
Male	56	(66.66)	4	(50)	12	(70.58)	6	(100)
Female	28	(33.33)	4	(50)	5	(29.42)	0	(0)

In group A ‘Wholly joint families’ (*n*=40, 46.60%) and ‘Partially joint families’ (*n*=28, 33.33%) were common (Table 2). In group B also there were 3 cases of each in the wholly joint and partially joint families’ categories. Thus, the majority of patients attending the Psychiatry OPD and Geriatric Medicine OPD came from ‘Wholly joint families’ and ‘Partially joint families’. It signifies that in this study family jointness was adequate. On the contrary, in group C ‘Wholly nuclear families’ (*n*=13, 76.47%) were common and all patients in group D were from wholly nuclear families.

**Table 2:** Family jointness of patients in groups A, B, C and D

Types of families	Group A (n=84)		Group B (n=8)		Group C (n=17)		Group D (n=6)	
				
	*n*	%	*n*	%	*n*	%	*n*	%
Wholly nuclear	6	(7.14)	1	(12.5)	13	(76.47)	6	(100)
Partially nuclear	10	(11.95)	1	(12.5)	4	(23.52)	-	-
Partially joint	28	(33.33)	3	(37.25)	-	-	-	-
Wholly joint	40	(46.60)	3	(37.25)	-	-	-	-

For statistical analysis, patients were divided into two categories: (I) with poor and absent objective social support, and (II) with moderate and good objective social support. Chi-square test with Yate correction was applied to test the statistical significance. More than 50% of patients in all the study groups had moderate or good objective social support (Table 3a).

In group A (*n*=84), 30 patients (35.57%) had moderate, 19 patients (22.61%) had good, 6 patients (7.14%) had absent and 29 patients (34.52%) had poor objective social support. Out of 8 patients in group B, moderate and good objective social support was present in 3 (37.5%) and 2 patients (25%), respectively. Absent and poor objective social support was present in 1 and 2 patients, respectively. Table 3a shows that there were 54 patients (58.60%) out of the 92 patients in groups A and B in the ‘moderate’ and ‘good’ categories, but in groups C and D there were 21 patients (91.30%) out of 23. Thus, it was observed that a significantly higher percentage of patients in groups C and D reported good and moderate social support, and a significantly lower percentage of patients in groups C and D reported no or poor objective social support than patients in groups A and B. This observation is interesting as patients in groups C and D were living in an old age home whereas patients in groups A and B were living with their families (Table 3a).

**Table 3:** Objective and perceived social support among patients in groups A, B, C and D

	Group A (*n*=84)		Group B (*n*=8)		Group C (*n*=17)		Group D (*n*=6)	
				
	*n*	%	*n*	%	*n*	%	*n*	%
(a) Objective social support*								
*Category I*								
Absent	6	(7.14)	1	(12.5)	*-*	-	*-*	-
Poor	29	(34.52)	2	(25)	2	(11.76)	*-*	-
*Category II*								
Moderate	30	(35.57)	3	(37.5)	9	(52.29)	6	(100)
Good	19	(22.61)	2	(25)	6	(35.29)	-	-
**(b) Perceived social support**								
*Category I*								
Absent	14	(16.66)	1	(12.5)	-	-	-	-
Poor	45	(53.57)	4	(50)	-	-	-	-
*Category II*								
Moderate	23	(27.38)	2	(25)	9	(52.94)	6	(100)
Good	2	(2.38)	1	(12.5)	8	(47.05)	-	-

* χ^2^=7.25; p<0.01

Table 3b shows that in group A, 45 patients (53.57%) perceived their social support to be poor; another 14 patients (16.66%) perceived that they had no social support. Perceived social support was moderate or good in 23 (27.38%) and 2 cases (2.38%), respectively. In group B, a similar trend was seen. Poor perceived social support and absence of perceived social support were reported by 4 (50%) and 1 subject (12.5%), respectively. In contrast, in groups C and D (Mumukshu Bhavan residents) not a single patient reported perceived social support as absent or poor.

For statistical analysis, the findings of Table 3b were clubbed into two categories according to the level of perceived social support (I) poor and absent perceived social support in groups A and B, and (II) moderate and good perceived social support in groups C and D. It was noted that a majority of patients in group A (16.66% + 53.57%=70.23%) and in group B (12.5% + 50%=62.50%) either lacked or had poor perceived social support. In contrast, none of the patients selected from Mumukshu Bhavan had absent or poor perceived social support.

Table 3b shows that in category II, 28 (23+2+2+1=28) patients (30%) perceived social support to be either moderate or good whereas all (100%) patients of Mumukshu Bhavan reported either moderate or good perceived social support. To know whether this 100% is considerably higher and statistically significant than 30% or not, the Z-test was applied which revealed a Z-value of 14.65, which is highly significant (p<0.01). It appears that patients living in Mumukshu Bhavan had better perceived social support than those who were living with their family members.

Table 4 shows that depressive disorders, bipolar affective disorders and schizophrenia were common illnesses in psychiatric patients of group A. In group B, depressive disorder (*n*=5, 62.5%) was the most common illness.

In group C, there were 6 cases (35.28%) of major depressive disorder, 5 cases (29.41%) of dysthymic disorder and 3 cases (17.64%) of generalized anxiety disorder (GAD). One case each of somatization disorder, primary insomnia and dementia of Alzheimer type was present in each category.

Depressive disorder (*n*=2; 33.33%) and dementia of Alzheimer type (*n*=2; 33.33%) were the most common psychiatric illnesses in group D. Depressive disorder, anxiety disorder, primary insomnia and dementia of Alzheimer disease were present in 2 (33.33%), 1 (16.66%), 1 (16.66%) and 2 (33.33%) cases, respectively (Table 4).

**Table 4:** DSM-IV diagnosis of patients in groups A, B, C and D

		Group A		Group B		Group C		Group D	
					
		*n*	%	*n*	%	*n*	%	*n*	%
1.	Mood disorders								
	Depressive disorders								
	Major depressive disorder: Single episode	11	(13.09)	3	(97.50)	4	(23.55)	2	(33.33)
	Major depressive disorder: Recurrent	7	(8.33)	2	(25.00)	2	(11.76)	-	-
	Dysthymic disorder	6	(7.14)	-	-	5	(29.41)	-	-
	Depressive disorder NOS	1	(1.19)	-	-	-	-	-	-
2.	Bipolar disorders								
	Bipolar I disorder: Single manic episode	2	(2.38)	-	-	-	-	-	-
	Bipolar I disorder: Current episode manic	4	(4.76)	-	-	-	-	-	-
	Bipolar I disorder: Current episode depressed	1	(1.19)	-	-	-	-	-	-
	Bipolar II disorder: Current episode depressed	5	(5.95)	-	-	-	-	-	-
3.	Schizophrenia and other psychotic disorders								
	Schizophrenia	20	(23.80)	-	-	-	-	-	-
	Schizophreniform disorder	2	(2.38)	-	-	-	-	-	-
	Brief psychotic disorder	5	(5.95)	-	-	-	-	-	-
	Psychiatric disorder due to general medical condition	1	(1.19)	-	-	-	-	-	-
4.	Anxiety disorders								
	Obsessive-compulsive disorder	1	(1.19)	-	-	-	-	-	-
	Generalized anxiety disorder	4	(4.76)	1	(12.50)	3	(17.64)	1	(16.66)
5.	Somatoform disorders								
	Somatization disorder	-	-	-	-	1	(5.88)	-	-
	Conversion disorder	1	(1.19)	-	-	-	-	-	-
6.	Delirium and amnestic disorders								
	Amnestic disorder due to head injury	1	(1.19)	-	-	-	-	-	-
	Delirium due to general medical condition	1	(1.19)	-	-	-	-	-	-
7.	Dementia								
	Dementia of Alzheimer type	6	(7.14)	1	(12.50)	1	(5.88)	2	(33.33)
	Vascular dementia	2	(2.38)	-	-	-	-	-	-
8.	Sleep disorders								
	Primary insomnia	2	(2.38)	1	(12.50)	1	(5.88)	1	(16.66)
9.	Substance-related disorder								
	Alcohol dependence	1	(1.19)	-	-	-	-	-	-

NOS - not otherwise specified

Table 5 shows that in group A essential hypertension (13, 15.47%) was the most common co-morbid physical illness followed by cataract (8, 9.52%) and hearing impairment (8, 9.52%). In group C also, essential hypertension (3, 17.64%) was the most common physical illness. In group D, there was 1 case each of essential hypertension, cataract and hearing impairment.

**Table 5:** Pattern of associated physical illness in group A (*n*=84), group C (*n*=17) and in group D (*n*=6)

Diagnosis		Group A		Group C		Group D	
				
		*n*	%	*n*	%	*n*	%
1.	Essential hypertension	13	(15.47)	3	(17.64)	1	(16.66)
2.	Hearing impairment	8	(9.52)	2	(11.76)	1	(16.66)
3.	Cataract	8	(9.52)	2	(11.76)	1	(16.66)
4.	Paresis	1	(1.19)	-	-	-	-
5.	Diabetes mellitus	3	(3.57)	1	(5.88)	-	-
6.	Respiratory tract infection	1	(1.19)	-	-	-	-
7.	Severe dehydration	4	(4.76)	-	-	-	-
8.	Grand mal epilepsy	1	(1.19)	-	-	-	-
9.	Congestive heart failure	1	(1.19)	-	-	-	-
10.	Bronchial asthma	1	(1.19)	-	-	-	-
11.	Glaucoma	1	(1.19)	-	-	-	-
12.	Pulmonary tuberculosis	1	(1.19)	-	-	-	-
13.	Dermatitis	1	(1.19)	-	-	-	-
14.	Otitis media	1	(1.19)	-	-	-	-
15.	Benign prostatic hypertrophy	1	(1.19)	-	-	-	-
16.	Goitre	1	(1.19)	-	-	-	-
17.	Fracture	1	(1.19)	-	-	-	-
18.	Leprosy	1	(1.19)	-	-	-	-
19.	Prerenal azotaemia	1	(1.19)	-	-	-	-
20.	Abscess	1	(1.19)	-	-	-	-
21.	Corneal ulcer	1	(1.19)	-	-	-	-
22.	Pedal oedema	1	(1.19)	-	-	-	-
23.	Atherosclerotic heart disease	1	(1.19)	-	-	-	-
24.	Arthritis	2	(2.38)	1	(5.88)	-	-
25.	Myopathy	1	(1.19)	-	-	-	-

## DISCUSSION

In group A, 92.85% of the patients were in the age range of 60–69 years. Prasad *et al.,* Nandi *et al.* and Tiwari and Srivastava reported 74%, 87.6% and 59.70% cases, respectively, in the same age range.^1^,^19^,^20^ In the 70–79 years age range in group A, there were 5.95% cases. Nandi *et al.* and Tiwari and Srivastava reported 22.3% and 24.27% of patients, respectively, in 70–79 years age group.^1^,^20^

In the 80 years and above category, there was only 1case (1.19%) in our study. Nandi *et al.* and Tiwari and Srivastava reported 11.6% and 16.02% cases, respectively (Table 1).^1^,^20^

The study by Prasad *et al.* was a hospital-based study at NIMHANS, Bangalore and that of Nandi *et al.* and Tiwari and Srivastava were rural field surveys in West Bengal and Lucknow (UP), respectively. The differences in observation from that of Prasad *et al.* could be due to geographical differences and from that of Nandi *et al.* and Tiwari and Srivastava could be due to the fact that their studies were field surveys conducted in different geographical areas.^1^,^19^,^20^

In group A, the majority of patients (53.57%) perceived their social support as poor and another 16.66% felt that they had no social support Table 3b; 27.38% of group A patients perceived their social support as moderate and 2.38% as good. On the contrary, objective social support was moderate in 35.57% and good in 22.61% of group A patients (Table 3a); Thus, on this scale while 58.18% had moderate or good objective social support, 29.76% of patients perceived their social support to be moderate or good and 28.42% (58.18% – 29.76% = 28.42%) of patients did not perceive their social support to be moderate or good, though on the objective scale, they had moderate or good social support. Likewise, in group B also, 37.5% of patients perceived their social support be either moderate (25%) or good (12.5%), although 62.5% had moderate (37.5%) or good (25%) objective social support.

Thus, 25% (62.5% – 37.5% = 25%) of patients who had either good or moderate objective social support perceived it to be poor or absent.

All group A and group B patients were living with their relatives and were brought to the hospital by them for treatment. The observed discrepancy in objective and perceived social support could be attributed to (i) lack of care given to those patients at home; (ii) group A had 33.33% cases of psychosis, some of them had disturbed perception of their interpersonal relationships with their caregivers; (iii) some elderly patients’ expectation of care from their families was more than what their families could provide.

Surprisingly, all patients who were living in Mumukshu Bhavan (groups C and D) lived away from their families but they perceived their social support to be good or moderate.

The contrasting observations in groups A and B compared with groups C and D with respect to social support is an interesting observation. Individuals living in Mumukshu Bhavan were not living with their children's families and were dependent on an institution but they had a better perception of social support (Table 3). This difference could be due to the fact that they were living in a place where spiritual discourses, prayers in groups, etc. were regular features, which reduced their expectations and desires. They were people who had come to stay in Varanasi with a desire for moksha.

In group A, depressive disorder was seen in 29.76% of psychiatric patients. Venkobarao, Nandi *et al.* and Tiwari and Srivastava reported depressive disorder in 22.2%, 55.2% and 30.09% of geropsychiatric patients, respectively.^1^,^15^,^20^ In this study there were 8.69% cases of anxiety disorder. Venkobarao and Madhavan found 5.34% cases of anxiety disorder. Tiwari and Srivastava found 21.35% cases of anxiety disorder among geropsychiatric patients of rural Uttar Pradesh.^1^,^21^

Dementia was present in 9.52% of geropsychiatric patients of group A. Nandi *et al.* and Tiwari and Srivastava found a prevalence of 1.6% and 6.7% of dementia, respectively in their studies. In this study, there was a higher percentage of dementia cases among our patients. The difference may be due to the fact that they studied patients in the community.^1^,^20^ Findings of depressed mood and guilt feeling in this study were similar to those of Venkobarao (Table 4).^15^

The highest prevalence of geropsychiatric morbidity was found in sixth and seventh decades of life. Family jointness was adequate in the majority of patients. Objective social support was moderate for the majority of patients coming to hospital but perceived social support was poor.

Patients of Mumukshu Bhavan perceived better social support than those who were living with their children's families. Depressive disorder was the most common psychiatric illness followed by schizophrenia and bipolar disorder. Associated physical illness was found in 61.73% of patients and among them hypertension was the most common.

As geriatric patients with all types of psychiatric illness were included, it would have been desirable to study a larger number of patients so that each diagnostic category had a large number of patients. This was, however, not possible due to constraints of time. Further studies with a larger sample focusing on elderly people coming from a particular socio-economic class (e.g. lower, middle and higher classes) may reveal specific geropsychiatric problems among each of these groups. With regard to perceived social support, the study needs to be replicated in existing old age homes.

Elderly people constitute our ‘guidance shell’ and we must do our best to protect our candle of light.
